# Understanding COVID-19 booster information seeking in a collectivist context: the roles of social expectations, trust in experts, and uncertainty

**DOI:** 10.3389/fpubh.2025.1611711

**Published:** 2025-07-15

**Authors:** Xiaoshan Austin Li, Katharine Hubbard, Jooyun Hwang

**Affiliations:** ^1^Department of Communication, Faculty of Humanities and Social Sciences, Beijing Normal-Hong Kong Baptist University, Zhuhai, China; ^2^Department of Mass Communication, Winthrop University, Rock Hill, SC, United States; ^3^Department of Journalism, Public Relations, and Advertising, Soongsil University, Seoul, Republic of Korea

**Keywords:** health communication, risk information seeking, trust in experts, uncertainty, subjective norms, COVID-19 booster

## Abstract

**Background:**

Effective public health communication relies on understanding how individuals seek information during health emergencies. While previous work has investigated vaccine hesitancy and acceptance, little is known regarding the psychological and social motivations behind COVID-19 booster information-seeking in collectivist societies.

**Objective:**

This study extends the Risk Information Seeking and Processing (RISP) model to explore the impact of trust in experts, risk uncertainty, and subjective informational norms on the public’s intention to seek information regarding COVID-19 booster shots in China.

**Methods:**

A national survey of 616 adults in China was undertaken. Structural equation modeling (SEM) examined hypothesized relationships among perceived advantages and disadvantages, affective responses, lack of information, trust in the expertise of others, uncertainty, perceived control over behavior, and social norms.

**Results:**

Informational subjective norms were the most significant predictor of intentions to seek information, indicating the influence of collectivist expectations on individual action. Trust in experts was positively associated with perceived risks and inversely related to perceived benefits—and decreased perceived information insufficiency. Uncertainty increased individuals’ perceived ability to gather and interpret information, but affective responses had limited direct effects.

**Conclusion:**

Findings highlight the need to incorporate social norms, trust relationships, and uncertainty management into public health education campaigns to support vaccine promotion. This study offers empirical evidence for designing culturally adaptive communication interventions that promote booster uptake among collectivist societies and comparable environments.

## Introduction

The COVID-19 pandemic underscored the critical role of risk communication in shaping vaccine behaviors. While much of the existing vaccine acceptance and hesitancy research has focused on individual-level psychological factors [e.g., (1)], far less attention has been paid to how cultural norms and political structures influence how people seek health-related risk information. There is limited understanding of how collectivist cultural values shape information behaviors in health systems where communication is tightly aligned with state narratives. Among many, China is a rich case for advancing this understanding.

Despite achieving high initial COVID-19 vaccination rates through centralized policy enforcement, the country experienced a decline in booster uptake following shifts in public health messaging and policy relaxation. This dynamic environment offers a unique opportunity to explore how individuals in a collectivist society respond to evolving public health recommendations regarding their motivation and capacity to seek credible information amid uncertainty.

To investigate these dynamics, we draw on the original Risk Information Seeking and Processing (RISP) model (2) but incorporate additional sociocultural and psychological variables relevant to the Chinese context. Based on a national survey of 616 adults in China, we examine how informational subjective norms, institutional trust, uncertainty, and affective responses shape individuals’ intentions to seek information about COVID-19 booster shots.

This study makes three key contributions to the literature. First, it suggests that collectivist social expectations may override emotional responses as primary motivators for health information seeking. Second, it reveals the paradoxical role of trust in experts in state-aligned health systems, in which trust may both elevate perceived risk and reduce perceived benefit. Third, it shows that uncertainty, rather than weakening informational control, may enhance individuals’ confidence in navigating health information.

Although situated in the Chinese context, our findings have broader implications for public health education and vaccine promotion strategies in other collectivist or state-aligned systems across Asia and globally. They offer insights for international health organizations aiming to tailor public communication to fit diverse governance environments in future public health emergencies.

## Literature review

The Risk Information Seeking and Processing (RISP) Model and Key Constructs.

The present study adopts the Risk Information Seeking and Processing (RISP) model (2), which draws heavily from the theory of planned behavior (3) and the heuristic-systematic model (4) to explain how social and cognitive factors interact to shape COVID-19 booster information seeking in China.

### Information insufficiency, risk and benefit perceptions, and affective responses

Central to the model is information insufficiency, the perceived gap between current and desired knowledge, which motivates individuals to seek information to close the gap. Meanwhile, informational subjective norms (the perceived social pressures individuals feel to stay informed about risks) also critically influence an individual’s motivation to seek information, particularly in collectivist cultures such as China (5). The model also incorporates perceived information-gathering capacity, which reflects an individual’s belief in their ability to find and understand relevant information, as well as relevant channel beliefs or perceptions regarding the credibility of information sources. Finally, the RISP model acknowledges the importance of affective responses, such as worry or anger, as emotional reactions to risks that can drive information-seeking behavior.

Emotions such as fear and anxiety are critical mediators of risk perception and behavior (6, 7). For instance, fear amplifies risk appraisals and motivates protective actions (7), while anger may reduce perceived vulnerability (6). During prolonged crises like COVID-19, however, repeated exposure to threat messages can lead to emotional fatigue, diminishing the motivational impact of affective responses over time (8, 9). This may explain why affective responses in our study showed limited direct effects on information-seeking intentions. Cultural norms in collectivist societies like China may further suppress individual emotional expression in favor of socially sanctioned behaviors (10), redirecting motivation toward conformity with group expectations rather than personal feelings.

In addition, RISP emphasizes the role of risk perception, an individual’s subjective assessment of the potential harm or danger associated with a situation or behavior (11). Higher perceived risk typically increases information-seeking as individuals attempt to reduce uncertainty and make informed decisions (2, 12). Theoretical frameworks like Prospect Theory (13) further illuminate how individuals evaluate risks and benefits under uncertainty. Prospect Theory posits that people are more sensitive to potential losses than gains (loss aversion), and their decisions often depend on how choices are framed (e.g., as gains or losses).

In COVID-19 booster uptake, individuals may weigh perceived risks (e.g., side effects) more heavily than perceived benefits (e.g., immunity), particularly when public health messaging emphasizes potential harms. Such framing effects could amplify information-seeking intentions as individuals strive to mitigate losses (11, 12). This aligns with the RISP model’s emphasis on risk perceptions as a driver of information insufficiency while offering a complementary lens for understanding how cultural or institutional framing of risks shapes decision-making.

Conversely, benefit perception refers to beliefs about the vaccine’s effectiveness in 3preventing infection (14). While earlier studies suggest that greater perceived benefits might encourage individuals to conform to positive beliefs (15), recent evidence indicates that higher perceived benefits may also lead to perceptions of sufficient existing knowledge (16). Thus, we propose:

*H1a*: Perceived risks will be positively associated with information insufficiency regarding COVID-19 booster shots.

*H1b*: Benefit perception will be negatively associated with information insufficiency regarding COVID-19 booster shots.

These perceptions are closely related to corresponding affective responses. Negative affective responses (e.g., fear, worry) often drive information seeking to reduce uncertainty (17, 18). Meanwhile, positive affective responses (e.g., hope, optimism) may encourage a more thorough information search (19). Accordingly:

*H2a*: Perceived risks related to COVID-19 booster shots will be positively associated with negative affective responses.

*H2b*: Benefit perception to COVID-19 booster shots will be positively associated with positive affective responses.

*H3a*: Negative affective responses will positively influence the information-seeking intention about COVID-19 booster shots.

*H3b*: Positive affective responses will be positively associated with information-seeking intention related to COVID-19 booster shots.

Given this possibility of differential effects of positive and negative emotional responses, the following research question arises:

RQ1: Which one of the affective responses (negative vs. positive) has a stronger influence on information-seeking intention regarding COVID-19 booster shots in China?

### Informational subjective norms

Complementing information insufficiency, the RISP model emphasizes the importance of social pressures to stay informed about a given risk (2). There are two types of subjective norms: (1) injunctive norms, which is the belief of what others think one should do, and (2) descriptive norms, referring to the perceptions of what others do (20). Informational subjective norms are specific to risk information seeking, as they depict one’s perception of social expectations regarding what they should know about a given risk.

Empirical studies confirmed that the country’s collectivist culture and the government’s emphasis on public health measures may amplify informational subjective norms. According to Lin et al. (21) and Liu et al. (22), social norms are a reliable predictor of vaccine acceptance among Chinese adults. These findings highlight the applicability of normative influences in health decision-making. We thus propose:

*H4*: Informational subjective norms will positively affect information-seeking intentions regarding COVID-19 booster shots.

### Perceived informational behavior control

The RISP model incorporates perceived information-gathering capacity, which reflects an individual’s belief in their ability to seek and comprehend relevant information. This concept is analogous to perceived behavioral control (PBC) in the Theory of Planned Behavior (3), from which the RISP model has its foundational reference. In the context of risk information seeking, perceived behavior control either facilitates or inhibits information-seeking behavior, depending on the individual’s self-efficacy in gathering information (2).

In their meta-analysis, (23) extrapolated that perceived behavior control consistently influences intentions in various health contexts. Additionally, people with high perceived capacity tend to feel they are more capable of understanding complex health information, interpreting scientific evidence, and making informed decisions about health-related behavior (24), whereas those with lower perceived capacity might feel overwhelmed by abundant information and struggle to determine reliable sources (25, 26).

The COVID-19 situation in China was both complex and rapidly changing, and perceived behavior control might be crucial in shaping individuals’ information-seeking behaviors regarding booster shots. Based on the documented importance of perceived behavioral control, we propose:

*H5*: Perceived seeking control will positively affect information-seeking intentions regarding COVID-19 booster shots.

### Extending the RISP model: trust and uncertainty

#### Trust in experts

Trust in health-related settings is often conceptualized as “the optimistic acceptance of a vulnerable situation in which the truster believes that the trustee will care for the truster’s interests” [(27), p. 615]. Following Hendriks et al.’s (28) work, the present study conceptualizes the trustworthiness of experts as the totality of expertise (e.g., knowledge), integrity (e.g., honesty), and benevolence (e.g., good intention).

The Trust, Confidence, and Cooperation (TCC) model (29) suggests that trust in authorities and experts is a key determinant of risk perception and acceptance in risk communication (11, 30). Specifically, lower trust in information sources may heighten risk perception and reduce perceived benefits, leading to unfavorable judgments toward recommended behaviors (31–33).

Trust in experts is not merely a function of perceived competence but also institutional credibility and alignment with societal values (34, 35). During the COVID-19 pandemic, institutional trust became a cornerstone of compliance with public health measures, particularly in state-aligned systems like China (36, 37). However, trust can be paradoxical: while it reduces skepticism toward institutional directives (35), it may also heighten scrutiny of expert warnings about risks (38). For example, transparent communication about vaccine uncertainties could inadvertently elevate risk perceptions while fostering trust (36). This duality underscores the need to disentangle trust’s role in shaping risk–benefit perceptions and affective responses within centralized health systems.

Studies have shown that people who trust experts may also be more aware of the uncertainties and risks associated with boosters, which leads to heightened perceptions of risk (39, 40). This may be due to experts being transparent about the evolving scientific knowledge or the potential for adverse outcomes (41, 42). Alternatively, trust in experts may also make individuals more critical or cautious, thus perceiving fewer benefits from the booster shots when new scientific data or advice creates uncertainty (28, 43).

Consequently, while trust in experts plays a crucial role in shaping one’s health decisions, it may also increase individuals’ awareness and skepticism about the benefits of specific health interventions. From these findings, we hypothesize that:

*H6a*: Trust in experts will be negatively associated with the perceived benefits of COVID-19 booster shots.

*H6b*: Trust in experts will be positively associated with the perceived risks of COVID-19 booster shots.

In various health-related contexts, trust in experts functions as a protective mechanism against uncertainty to alleviate negative emotions such as anxiety. When individuals perceive experts as competent, honest, and acting in the public’s best interests, they are less prone to feelings of fear or worry (44). Studies on public health crises have shown that higher trust in experts reduces worry or anxiety (45, 46). Based on the evidence:

*H6c*: Trust in experts will be negatively associated with negative affective responses regarding COVID-19 booster shots.

In addition, trust in experts may influence how individuals respond to social pressure or expectations. Ho et al. (47) demonstrated that trust in scientific authorities strengthens the influence of social norms on science-related behaviors, while more recent studies (48, 49) showed trust in experts amplifies how people respond to social expectations edge. In other words, when people trust experts more, they are more likely to behave according to social expectations. This interaction between trust and subjective norms leads us to ask:

RQ2: Will trust in experts moderate the relationship between informational subjective norms and information insufficiency, such that higher trust in experts will strengthen the positive association between informational subjective norms and information insufficiency?

#### Uncertainty

Uncertainty arises when “situations are ambiguous, complex, unpredictable or probabilistic; when information is unavailable or inconsistent; and when people feel insecure in their state of knowledge or the state of knowledge in general” [(50), p. 478]. In healthcare, there are several forms of uncertainty, such as uncertainty relating to the effect of treatments or prevention measures, personal vulnerability, and possible side effects that one can suffer (51). Such continuously changing preventive measures against the variants of SARS-CoV-2 overloaded the public and thus accelerated the development of the so-called ‘infodemic,’ making information searching even harder (52–56).

In contexts with persistent uncertainty, like the COVID-19 pandemic, individuals may adapt by accepting uncertainty rather than seeking to eliminate it. Consequently, higher uncertainty might paradoxically reduce perceived information insufficiency as individuals accept that complete knowledge is unattainable.

While RISP traditionally assumes information seeking reduces uncertainty, uncertainty management theory provides a complementary perspective where individuals may strategically manage rather than eliminate uncertainty (50, 57–59). When faced with high levels of uncertainty, individuals may experience heightened affective responses, such as worry or anxiety, that can motivate them to seek more information (50, 61).

*H7a*: levels of uncertainty will be negatively associated with information insufficiency.

Meanwhile, high levels of uncertainty often make individuals more attuned to social norms and environmental cues to determine the appropriate course of action. In situations with high uncertainty, individuals tend to rely more on the expectations of society or societal pressures, especially in a collectivist culture like China. In these situations, collective norms can influence personal conduct such that social expectations by peers, communities, or health authorities can significantly impact individuals’ motivation toward information.

*H7b*: levels of uncertainty will be positively correlated with informational subjective norms.

Under uncertainty, individuals may feel less control due to ambiguity or become more proactive in seeking trusted sources. This adaptation may increase their perceived control. The ability to access, understand, and utilize information, despite ambiguities, can help bolster one’s self-efficacy regarding decision-making. Therefore, rather than feeling overwhelmed, they might consider themselves better equipped to handle health-related decisions at times of great uncertainty. Thus, we hypothesize:

*H8*: Uncertainty will be positively associated with perceived seeking control regarding information seeking about COVID-19 booster shots.

Furthermore, levels of uncertainty may influence one’s perceptions of the benefits and risks associated with COVID-19 booster shots. For instance, a heightened level of perceived uncertainty may make people more sensitive to potential risks while diminishing potential benefits, which may impact their confidence in decision-making. This begets the following hypotheses:

*H9a*: Perceived uncertainty will be negatively associated with the perceived benefits of COVID-19 booster shots.

*H9b*: Perceived uncertainty will be positively associated with perceived risks of COVID-19 booster shots.

Due to model complexity and sample constraints, hypotheses H7 through H9 are treated as exploratory. These hypotheses aim to offer preliminary insights into the broader influence of uncertainty on informational subjective norms, perceived control, and risk–benefit appraisals. Confirmatory testing of these pathways would require larger and more diverse samples in future studies.

**Figure 1:**
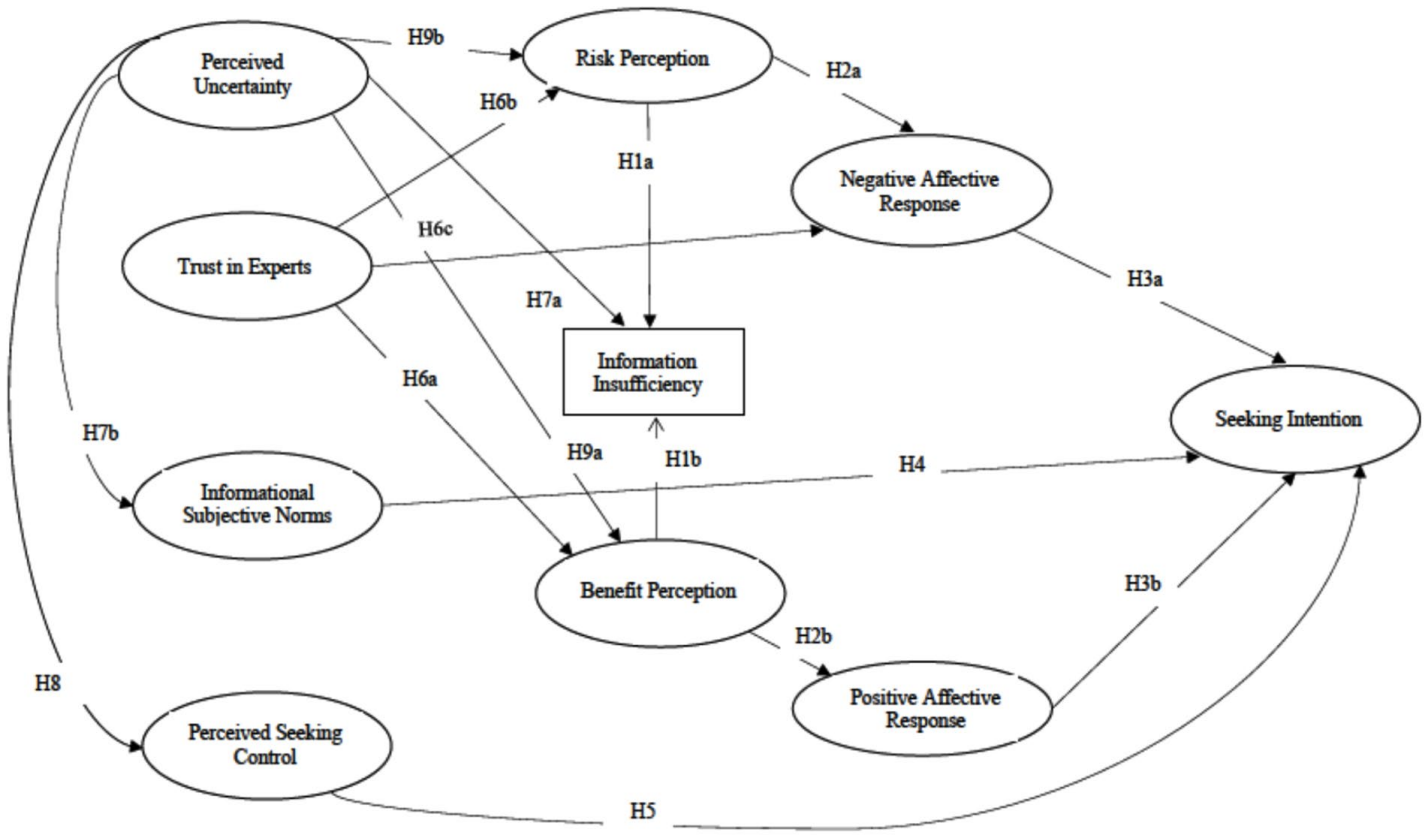
Hypothesized paths.

While the hypotheses (see Figure 1) address direct relationships between key variables, the complex nature of risk information-seeking behavior suggests that meaningful indirect relationships may be at play. The current study aims to capture these complex relationships, providing a comprehensive framework for understanding how various factors, particularly trust in experts and perceived uncertainty, influence information-seeking intention about COVID-19 booster shots.

## Materials and methods

### Study design

An online survey was conducted in China using panel members recruited from a prominent Chinese survey platform with three million registered and representative participants [e.g., (60)]. We adapted the questionnaire to Chinese, seeking insights from 10 Chinese native speakers who possessed doctoral degrees in various disciplines but were not part of this study. Several modifications were made upon receiving their feedback to improve the questionnaire’s precision and flow. Cronbach’s alpha values (greater than 0.70) suggested satisfying internal reliabilities.

The recruitment process for the survey involved two key criteria: participants had to be Chinese citizens aged 18 or older. Of the 735 collected responses, 119 were deemed invalid for the final analysis because they either completed the survey remarkably quickly or failed the attention-check questions.

### Participants

The final sample (*N* = 616) was comprised of 323 females (52.4%) and 293 males (47.6%). Participants’ mean age was 31.5 years old (*SD* = 6.5), ranging between 18 and 80 years old. Most participants had bachelor’s degrees and lived in a metropolitan area while working full-time (see Table 1). In addition, all participants had received COVID-19 vaccines, but only 34.6% reported receiving at least one booster shot.

**Table 1:** Demographic information.

		*N* (%)
Age	Mean = 31.53 years old	
Gender	Male	293 (47.6)
	Female	323 (52.4)
Education	High school	20 (3.2)
	Some college or an associate’s	66 (10.7)
	Bachelor’s degree	486 (78.9)
	Graduate degree	44 (7.1)
Area	Rural	18 (2.9)
	Municipal	158 (25.6)
	Metropolitan	413 (67)
	Suburb	27 (4.4)
Income	≤5,000	98 (15.9)
	5,000–10,000	260 (42.2)
	10,000-15,000	185 (30)
	≥ 15,000	73 (11.9)
Employment	Part-time	12 (1.9)
	Full-time	546 (88.6)
	Unemployed	58 (9.5)

### Measures

The study primarily deployed the well-cited measurements from the Risk Information Seeking and Processing Model [RISP; (2)]. All variables were assessed on a 5-point Likert scale, except where otherwise noted. While all variables were latent, perceived information insufficiency was treated as an observed variable. We performed confirmatory factor analyses (CFA) for all latent variables and removed items that scored factor loadings below 0.60 (62).

#### Untrustworthiness of health experts

Although our literature review centers on the perceived trustworthiness of experts, we operationalize the concept using Hendriks et al.’s (28) validated scale of the perceived untrustworthiness of scientists (e.g., whether they are irresponsible) on a 7-point Likert scale. To further clarify, respondents were asked to indicate their level of trust in several types of experts, including public health officials, scientists, and governmental health agencies (e.g., CDC China). While the term ‘experts’ is used generically in the analysis, the items reflect institutional affiliations relevant to the Chinese context. All items were reverse-coded so that higher scores reflect higher trust in experts. This approach ensured that higher composite scores reflect greater trust rather than untrustworthiness (*M* = 5.94, *SD* = 1.06, Cronbach’s *α* = 0.94).

#### Perceived uncertainty

The Uncertainty Management Theory suggests that severity, susceptibility, and self-efficacy are associated with one’s perceived medical uncertainty (63). Severity and susceptibility also shape one’s health-related behavior, such as information seeking (64). Therefore, we measured perceived uncertainty by asking participants to what extent they were uncertain about the severity of and susceptibility to the risks of COVID-19 boosters on a 7-point Likert scale (*M* = 4.81, *SD* = 0.86, Cronbach’s *α* = 0.70).

#### Seeking-related subjective norms

Participants rated their agreement (1 = strongly disagree, 5 = strongly agree) on four items (e.g., “Most of my family whose opinions I value expect me to seek information about the risks posed by receiving COVID-19 booster doses”). The items were averaged to create a composite, with higher scores representing higher normative expectancy for booster-related information seeking (*M* = 3.88, *SD* = 0.684, Cronbach’s *α* = 0.80).

#### Perceived seeking control

To measure the construct, participants rated their agreement (1 = strongly disagree, 5 = strongly agree) on four items (e.g., “I know where to look for information about the risks of receiving COVID-19 booster shots”). We created a composite by averaging the four items; a higher composite score reflects better control (*M* = 3.98, *SD* = 0.62, Cronbach’s α = 0.74).

#### Risk and benefit perceptions

Risk perceptions are often captured as the perceived likelihood of the risk occurring and the perceived seriousness of the risk if it were to happen. We measured risk likelihood with four questions, “how likely is it that you will be impacted by the potential risks posed by COVID-19 booster shots,” “If you were impacted by the potential risks posed by COVID-19 booster shots, how serious would that impact be,” “how likely is it that society will be impacted by the potential risks posed by COVID-19 booster doses,” and “if society were impacted by the potential risks posed by COVID-19 booster doses, how serious would that impact be?.” Response options were on a 5-point Likert scale ranging from “1 = not at all likely/serious” to “5 = extremely likely/serious.” The items were averaged to create a composite, with higher scores representing higher risk perceptions (*M* = 3.12, *SD* = 1.26, Cronbach’s *α* = 0.90).

In addition, measurements for benefit perception were adapted from the work of Lin et al. (54) and Sun et al. (65) to better align with the context of COVID-19 booster shots. Respondents were asked, “How beneficial will COVID-19 booster shots be for you personally,” and “How beneficial will COVID-19 booster shots be for society?” on a 5-point Likert scale ranging from “1 = not at all beneficial” to “5 = extremely beneficial (*M* = 4.28, *SD* = 0.65, Cronbach’s *α* = 0.72).

#### Affective risk responses

We intended to stay consistent with previous negative and positive affective response measurements. For negative affective responses, we based our measures on Witte’s (66) fear appeals but modified them to five items: “When I think about COVID booster shots, I get… frightened,” “…tense,” “…nervous,” “…anxious,” and “…uncomfortable.” Participants rated their agreement with these statements on a 7-point scale (1 = strongly disagree, 7 = strongly agree). Items were averaged to create a composite, with higher scores representing more negative responses toward the booster shots (*M* = 2.7, *SD* = 1.28, Cronbach’s *α* = 0.91). When assessing positive affective responses on a 7-point scale (1 = strongly disagree, 7 = strongly agree), we modified Watson et al.’s (67) work and asked respondents, “When I think about COVID booster shots, I feel… hopeful,” “…enthusiasm,” “…relieved,” and “…confident.” We then averaged the scores to create a composite (*M* = 5.5, *SD* = 0.94, Cronbach’s α = 0.86).

#### Perceived knowledge insufficiency

This concept describes the discrepancy between a person’s current knowledge level and the perceived level they think (arithmetic difference between current and needed knowledge) to align with prior RISP studies (2). While latent variable modeling was considered, this approach ensured consistency with the model’s original formulation. To measure this difference, we first asked, “How much do you currently know about the risks/benefits posed by COVID-19 booster doses?” and “How much do you need to know about the risks/benefits posed by COVID-19 booster doses?” on a scale of 0 to 100 (“0 = nothing” to “100 = all there is to know.”). We then calculated perceived knowledge insufficiency by subtracting the current knowledge score (*M* = 68.35, *SD* = 17.38) from perceived need (*M* = 67.02, *SD* = 19.62) to identify the extent to which participants’ current knowledge meets or falls short of perceived needs (*M* = −1.33, *SD* = 23.41).

#### Information seeking intent

Information-seeking intent was measured with four items on a Likert-type scale ranging from “1 = strongly disagree” to “5 = strongly agree” (e.g., “I will try to seek information about the risks posed by COVID-19 booster doses in the next 6 months),” “I will look for information about the risks posed by COVID-19 booster doses in the next 6 months,” (Kahlor, 2010). Items were averaged to create a composite, with higher values reflecting stronger intentions to seek information about COVID-19 booster shots (*M* = 3.98, *SD* = 0.7, Cronbach’s *α* = 0.85).

### Data analysis

We performed structural equation modeling (SEM) in *Mplus* 8.3 to validate the proposed model and relationships. SEM was selected for its ability to model latent variables (e.g., trust, uncertainty) and test complex mediation pathways simultaneously, which aligns with our goal of examining direct and indirect relationships posited by the extended RISP model (68). A maximum likelihood robust estimator was employed to combat multivariate normality issues. We conducted Mardia’s multivariate skewness and kurtosis tests, which indicated slight departures from normality; however, the use of robust estimation (MLR) addressed these concerns. In line with established guidelines, we also reported multiple fit indices. Numerous researchers, such as Bollen and Long (68) and Holbert and Stephenson (69), have suggested the disclosure of various model fit indicators, including the Chi-Square Test of Model Fit (χ2), the Root Mean Square Error of Approximation (RMSEA) with threshold values at or below 0.08, the Comparative Fit Index (CFI) and Tucker-Lewis Index (TLI), both requiring values at or above 0.90, as per Bentler and Bonett (70) and Hu and Bentler (71), and the Standardized Root Mean Square Residual (SRMR) with preferred values at or under 0.09.

## Results

We first verified the measurement model, which showed a good fit: *χ*2 (474) = 676.48(*p* < 0.001), RMSEA = 0.037 (90% CIs [0.031, 0.043]), CFI = 0.962, TLI = 0.958, SRMR = 0.045. We then added the proposed paths to test the structural model fit. Our results showed that the proposed model fits the data well: *χ*2 (678) = 1096.732 (*p* < 0.001), RMSEA = 0.045 (90% CIs [0.04, 0.05]), CFI = 0.932, TLI = 0.925, SRMR = 0.081.

**Figure 2:**
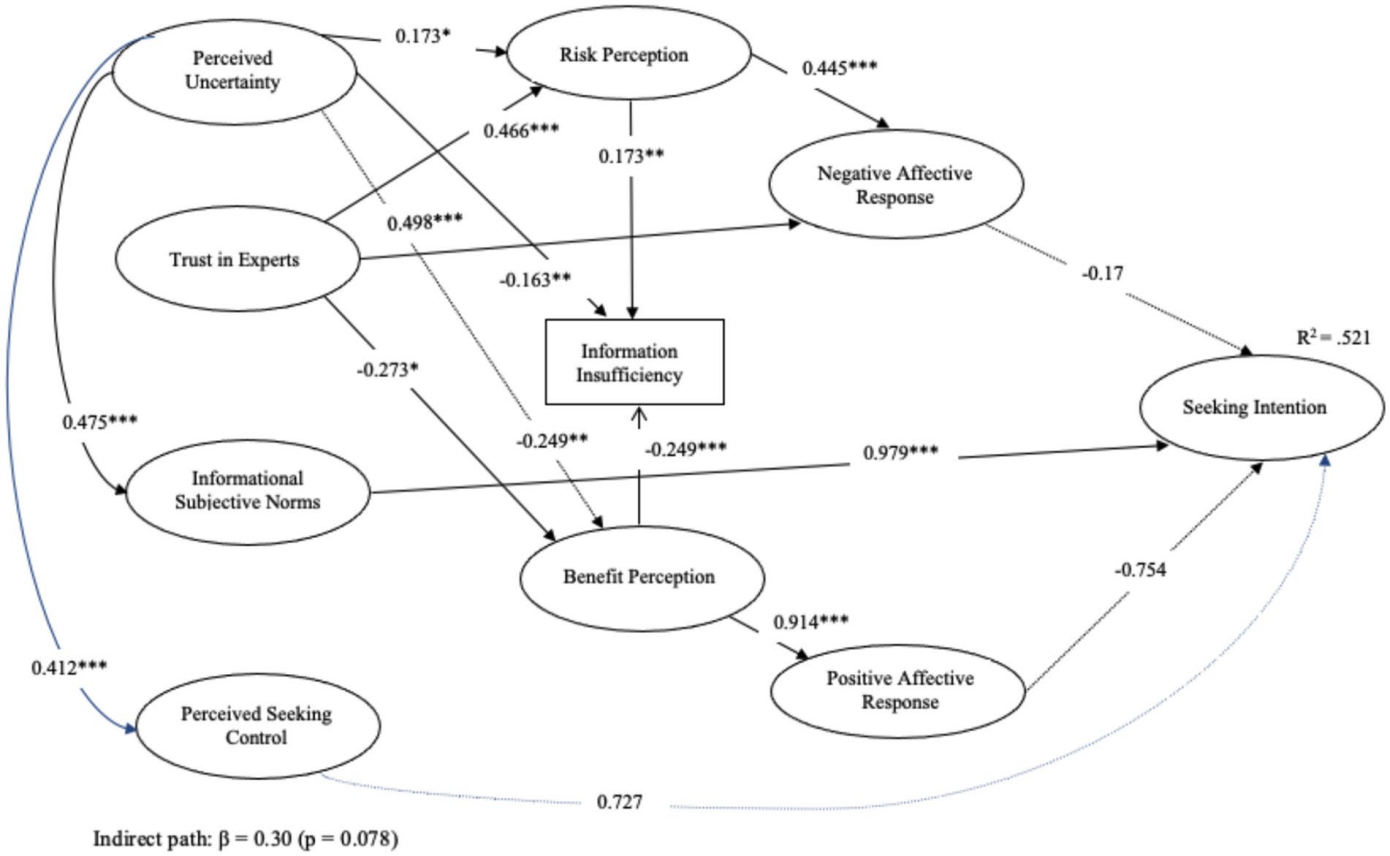
Dotted lines represent insignificant paths. The blue arrows indicate the mediation pathway explicitly tested (Uncertainty → Perceived Seeking Control → Seeking Intention; indirect path: *β* = 0.30, *p* = 0.078) * *p* < 0.05. ** *p* < 0.01. *** *p* < 0.001.

Figure 2 illustrates the structural model, with social norms (*β* = 0.979, p < 0.001) exerting the strongest effect on information-seeking intentions. At the same time, affective responses showed no significant influence (H3a/H3b), and the analysis revealed several significant relationships among the hypothesized paths, with a majority being supported. Regarding perceived knowledge insufficiency, both perceived risks (H1a: *β* = 0.173, *p* < 0.05) and perceived benefits (H1b: *β* = −0.249, *p* < 0.01) significantly predicted information insufficiency, supporting H1a and H1b. The relationships between perceptions and affective responses were also supported, with perceived risks positively predicting negative affective responses (H2a: *β* = 0.445, *p* < 0.001) and perceived benefits strongly predicting positive affective responses (H2b: *β* = 0.914, *p* < 0.001).

However, contrary to expectations, neither negative affective responses (H3a: *β* = −0.17, *p* = 0.17) nor positive affective responses (H3b: *β* = −0.754, *p* = 0.346) significantly predicted information-seeking intentions, thus failing to support H3a and H3b. Informational subjective norms emerged as a strong predictor of information-seeking intentions (H4: *β* = 0.979, *p* < 0.001), supporting H4. This large standardized coefficient indicates that each one-unit increase in subjective norms corresponds to a nearly one standard deviation increase in information-seeking intentions, underscoring the practical importance of collective expectations as a key motivator in health communication contexts. Additionally, perceived behavioral control showed only a marginally significant effect (H5: *β* = 0.727, *p* = 0.093).

Moreover, trust in experts was negatively associated with perceived benefits (H6a: *β* = −0.273, *p* < 0.05) but was positively associated with perceived risks (H6b: *β* = 0.466, *p* < 0.001), supporting H6a, H6b. Although a significant relationship exists between trust in experts and negative affective responses (H6c: *β* = 0.498, *p* < 0.001), the relationship is opposite to what was hypothesized. Thus, H6c was not supported.

In terms of the role of uncertainty in the model, the results revealed several significant relationships across pathways. Uncertainty was negatively associated with perceived knowledge insufficiency (H7a: *β* = −0.163, *p* < 0.01) and positively associated with both informational subjective norms (H7b: *β* = 0.475, *p* < 0.001) and perceived behavioral control (H8: *β* = 0.412, *p* < 0.001). Additionally, perceived uncertainty significantly predicted both perceived benefits (H9a: *β* = −0.249, *p* < 0.01) and perceived risks (H9b: *β* = 0.173, *p* < 0.05), supporting H9a and H9b. As noted earlier, we positioned H7 through H9 as exploratory to offer initial insights into the broader influence of uncertainty on social norms, perceived control, and perceptions of risk and benefit.

While these paths were supported by theory, they require further confirmation using larger and more diverse samples. Subgroup analyses confirmed the robustness of subjective norms as the primary predictor of information-seeking intentions. The effect remained stable across urban (*β* = 0.62, *p* < 0.001) and rural (*β* = 0.61, *p* < 0.001) populations, as well as lower-income (*β* = 0.58, *p* < 0.001) and higher-income (*β* = 0.62, *p* < 0.001) groups. Trust in experts, however, showed no significant association with intent in any subgroup (urban: *β* = −0.11, *p* = 0.105; rural: *β* = −0.05, *p* = 0.218).

In addition, the first research question (RQ1) intended to explore which type of affective response had a more substantial influence on information-seeking intentions. The results indicated that while neither type of affective response significantly affected seeking intent, positive affective responses showed a stronger relationship (*β* = −0.754, *p* = 0.346) than negative affective responses (*β* = −0.17, *p* = 0.17). As to the second research question (RQ2), which investigated whether trust in experts moderated the relationship between informational subjective norms and information insufficiency, the result revealed a non-significant moderation effect (*β* = 0.30, *p* = 0.17). Specifically, neither trust in experts (*β* = −0.502, *p* = 0.231) nor informational subjective norms (*β* = −0.328, *p* = 0.408) directly affected information insufficiency. The interaction between trust and informational subjective norms was also non-significant (*β* = 0.297, *p* = 0.171). These findings suggest that trust in experts does not moderate the relationship between informational subjective norms and information insufficiency (Table 2).

**Table 2:** Model statistics.

Hypothesis	Path	*β*	SE	*p*
H1a	Risk Perception → Perceived Knowledge Insufficiency	0.173	0.062	< 0.05
H1b	Benefit Perception → Perceived Knowledge Insufficiency	−0.249	0.091	< 0.01
H2a	Risk Perception → Negative Affect	0.445	0.045	< 0.001
H2b	Benefit Perception → Positive Affect	0.914	0.043	< 0.001
H3a	Negative Affect → Seeking Intent	−0.170	0.065	0.170
H3b	Positive Affect → Seeking Intent	−0.754	0.089	0.346
H4	Seeking Norms → Seeking Intent	0.979	0.086	< 0.001
H5	Seeking Control → Seeking Intent	0.727	0.093	0.093
H6a	Trust → Benefit Perception	−0.273	0.049	< 0.05
H6b	Trust → Risk Perception	0.466	0.050	< 0.001
H6c	Trust → Negative Affect	0.498	0.051	< 0.001
H7a	Perceived Uncertainty → Perceived Knowledge Insufficiency	−0.163	0.060	< 0.01
H7b	Perceived Uncertainty → Seeking Norms	0.475	0.049	< 0.001
H8	Perceived Uncertainty → Seeking Control	0.412	0.053	< 0.001
H9a	Perceived Uncertainty → Benefit Perception	−0.249	0.050	< 0.01
H9b	Perceived Uncertainty → Risk Perception	0.173	0.054	< 0.05

## Discussion

### Theoretical and practical implications

The present study extends our understanding of risk information-seeking behavior by examining how three key factors—social norms, trust in experts, and uncertainty—shape information-seeking in China’s collectivist context, with implications for global health communication practices. Drawing upon the Risk Information Seeking and Processing (RISP) model (2), the current research makes significant contributions to risk communication by (1) examining both positive and negative affective responses in risk information seeking, (2) testing the impact of trust in experts in the context of a collectivist culture; and (3) exploring the effect of uncertainty on various paths to information seeking.

### Risk and benefit perceptions in a framing context

Our analysis reveals key relationships central to the study’s research questions. First, both risk and benefit perceptions, albeit in opposite directions, significantly influenced information insufficiency: while risk perceptions increase perceived knowledge gaps, benefit perceptions decrease them. This pattern aligns with established risk communication literature that suggests risk and benefit perceptions often have opposing effects on risk-related behaviors (11, 72). These findings indicate that health communicators should balance messages of risk and benefit, emphasizing benefits that might reduce motivation for further information seeking while highlighting risks that might stimulate it. Critically, how individuals interpret these messages may depend on their trust in the institutions or experts delivering them.

Importantly, individuals’ interpretation of these framed messages is often shaped by their trust in institutional sources, especially in systems where public health messaging is tightly controlled. In such contexts, trust not only moderates message reception but also amplifies the salience of perceived losses over gains.

### The paradox of trust in experts

A dual pattern emerged about trust in experts, which was positively associated with risk perceptions and negatively associated with perceived benefits. This pattern aligns with Prospect Theory’s assertion that individuals prioritize loss avoidance under uncertainty (73). In the Chinese institutional context, trusted experts may frame booster-related risks (e.g., side effects) as losses to encourage compliance, which may inadvertently heighten individuals’ sensitivity to risk. Conversely, benefit perceptions may diminish if individuals perceive booster uptake as a ‘gain’ already secured through prior vaccination, therefore reducing the urgency to seek additional information. This framing dynamic highlights the interplay between institutional messaging strategies and Prospect Theory’s loss aversion principle.

Additionally, trust negatively predicted perceived benefits but positively predicted perceived risks and negative affective responses. These relationships counter traditional findings in risk communication, where trust in experts typically reduces risk perceptions and increases benefit perceptions (33, 74). Several factors might explain this unexpected pattern. First, the evolving nature of COVID-19 information may have affected how expert communications were perceived. When experts acknowledge uncertainties or change recommendations, it might simultaneously build trust through transparency while raising risk awareness (39, 75). Second, higher trust in experts might lead to greater attention to expert warnings about risks, thereby increasing risk perceptions rather than reducing them (38). Third, the institutional nature of expert communication during COVID-19 might have emphasized risks over benefits to promote preventive behaviors (42).

The positive association between trust in experts and risk perceptions contrasts with Western studies, where trust typically reduces risk appraisal (74). However, in state-aligned systems like China, institutional credibility may heighten attention to expert warnings about risks, as seen in (38) work on COVID-19 conspiracy beliefs. Transparent risk communication from trusted authorities may be associated with increased public vigilance, particularly when uncertainties persist (39). This suggests that trust in experts operates differently in centralized systems, where alignment between institutional and expert messaging reinforces risk sensitivity as a form of social responsibility. This dual effect likely reflects both a culturally embedded interpretation of institutional trust and a structural feature of risk communication in centralized systems, where expert messaging and policy alignment shape how risk and benefit are perceived.

#### Emotion, uncertainty, and cultural messaging in collectivist contexts

Our most striking finding contradicts core RISP assumptions: emotional reactions did not motivate information seeking about boosters, unlike in Western contexts. In contrast to the RISP model, affective responses did not drive information seeking. Instead, social expectations dominated decision-making. This reflects China’s cultural prioritization of communal harmony over individual concerns. In other words, social norms typically outweigh affective responses (10). For another, the dominance of social norms over affective responses aligns with institutionalized health communication in centralized systems, where collective compliance often supersedes individual emotions (76).

Additionally, China’s strong government guidance during the COVID-19 pandemic may have minimized the relevance of individual emotions in information-seeking decisions (38, 77). The consistency of subjective norms across geographic and socioeconomic subgroups underscores their primacy in collectivist health communication. While trust in experts is often theorized to drive compliance, its non-significance in our analyses suggests that institutional trust in China may function indirectly by reinforcing social conformity rather than directly motivating information-seeking behavior (38). This interpretation aligns with cultural frameworks where communal expectations supersede individual trust in authorities (78).

Results suggest that health communication strategies in collectivist contexts should focus on creating social consensus and institutional guidance about information-seeking behavior rather than appeals to emotions. In collectivist contexts, such as China, where trust in governmental and institutional expertise is generally high, individuals may perceive that their current knowledge, derived from official sources, is sufficient for making informed health decisions. This cultural context may reduce the likelihood of perceived knowledge gaps, as individuals may rely more heavily on institutional guidance rather than personal exploration to feel adequately informed (38, 78).

Moreover, the first RQ examined how positive and negative affective reactions influenced information-search intentions. The results showed that both types of affects failed to predict information search behavior significantly; however, positive affect was more closely related to this factor (*β* = −0.754, *p* = 0.346) than negative ones (*β* = −0.17, *p* = 0.17). This finding raises questions about traditional assumptions in risk communication that negative emotions are primary drivers of information seeking (12). The non-significant results for both affect types might suggest that in long-term health crises like COVID-19, emotional responses become less influential in driving information-seeking behaviors (77). In China, where state-aligned messaging consistently emphasizes collective resilience, individuals may have habituated to fear appeals, redirecting their focus toward normative expectations. Additionally, cultural norms prioritizing emotional restraint in collectivist societies (10) may suppress the role of personal affect in health decisions, further explaining the dominance of subjective norms over emotional drivers. Therefore, these results suggest that affective pathways in the RISP model might indeed be culturally bounded, particularly in collectivist contexts characterized by prolonged and institutionalized risk communication. These same cultural dynamics that muted emotional expression also appeared to influence how individuals processed uncertainty and managed information-seeking decisions.

The significant role of uncertainty in shaping both informational subjective norms and perceived behavioral control extends our understanding of how uncertainty influences information-seeking behavior. Our findings show that uncertainty positively predicts subjective norms (*β* = 0.475, *p* < 0.001) and perceived behavioral control (*β* = 0.412, *p* < 0.001), suggesting that under conditions of uncertainty, individuals may become more attuned to social cues while also feeling more capable of managing information-seeking behavior. This pattern aligns with uncertainty management theory (50), which suggests that individuals employ multiple strategies to cope with uncertainty. However, the positive relationship between uncertainty and perceived behavioral control is somewhat surprising, as previous research has often found uncertainty to decrease perceived control. This unexpected finding may be explained by the unique context of COVID-19 in China, where a robust information infrastructure and clear institutional guidance might have enhanced individuals’ confidence in their ability to find information despite underlying uncertainties (53). These findings indicate that public health organizations should not shy away from acknowledging uncertainties but rather combine such acknowledgments with clear guidance on information-seeking channels and resources, particularly in contexts with strong institutional support systems.

To further clarify how uncertainty influences information-seeking behavior, we investigated whether perceived behavioral control mediates the relationship between uncertainty and the intention to seek information. Results revealed a significant direct effect of uncertainty on control (*β* = 0.412, *p* < 0.001) and a marginally significant indirect effect on intentions through perceived behavioral control (*β* = 0.30, *p* = 0.078). Although marginally significant, this indirect effect (*β* = 0.30) suggests a practically meaningful mechanism, that is, institutional guidance appears capable of transforming uncertainty into actionable agency, enhancing perceived control by nearly one-third of a standard deviation, thereby promoting greater information-seeking. Though exploratory, this finding extends uncertainty management theory by illustrating how institutional infrastructure can transform ambiguity into agency, without altering our primary conclusion about normative dominance.

#### Social norms in collectivist health crises

RQ2 aimed to investigate whether trust in experts moderated the relationship between informational subjective norms and information insufficiency. Our analysis revealed that neither the main effects (trust: *β* = −0.502, *p* = 0.231; subjective norms: *β* = −0.328, *p* = 0.408) nor their interaction (*β* = 0.297, *p* = 0.171) significantly predicted information insufficiency, which suggests that the relationship between informational social norms and perceived knowledge insufficiency remains consistent regardless of trust levels. This result is particularly interesting in the Chinese context, where trust in authorities and social norms typically strongly influence behavior (78). One possible explanation is that trust and norms function as parallel rather than synergistic forces in institutionalized contexts. Since both derive from alignment with official narratives, their joint influence may be redundant. Alternatively, the high baseline trust in health authorities may have restricted variability, which limits the detection of interactive effects.

The lack of moderation reflects the institutionalized communication environment, where trust and norms operate independently rather than interactively (53). In state-aligned systems, institutional trust is deeply ingrained and functions as a stable heuristic for accepting official guidance, while social norms derive from collective compliance with centralized directives. This separation suggests that the expertise of experts does not amplify the effect of norms on knowledge gaps, as both are already aligned with institutional authority. Thus, health communication strategies should treat trust-building and social norm cultivation as separate but complementary approaches rather than assuming they will enhance each other’s effects.

Finally, the strong relationship between informational subjective norms and information-seeking intentions (*β* = 0.979, *p* = 0.001), together with only marginal effects of perceived behavioral control (*β* = 0.727, *p* = 0.093), suggests that social influences may be of significance in collectivist cultures facing health crises. This result aligns with recent research on COVID-19 communication in East Asian contexts, where social norms often play a dominant role in shaping health behaviors [e.g., (21)]. Thus, the implication is that health communication strategies in collectivist contexts should stress community participation and leverage social networks to promote information-seeking behaviors.

#### Limitations and future research

Firstly, our study relies on behavioral intentions rather than actual behaviors. This gap suggests that social-cognitive models are generally more accurate in predicting one’s intentions than actual behaviors [e.g., (78–82)]. In essence, it is essential to exercise caution when extending the study’s findings to explain individuals’ actual seeking behavior regarding COVID-19 booster shots. Therefore, future studies should consider closing the gap by incorporating additional variables, such as Ajzen (3).

Secondly, participants reported low perceived knowledge insufficiency, likely reflecting strong trust in institutional communication sources in China’s COVID-19 context. This unexpected finding suggests that in contexts with strong institutional messaging, people may feel adequately informed despite objective knowledge gaps. Future research could examine contexts where perceived information gaps are more pronounced, such as when conflicting information exists, or trust in institutions is lower.

Thirdly, while providing valuable insights, the generalizability of our study is limited due to the relatively homogeneous sample, which predominantly comprises young, educated individuals residing in urban areas. Although we conducted subgroup analyses across geographic and socioeconomic groups and found consistent patterns, these results should still be interpreted cautiously. For example, this demographic profile likely amplifies the observed role of subjective norms and institutional trust, given that urban and educated groups typically have greater digital access, higher institutional trust, and more exposure to official public health messaging in China. Consequently, our results might overstate the influence of norms and institutional trust while potentially underestimating barriers such as lower health literacy and reduced media access that rural or lower socioeconomic populations face. Future research should employ stratified sampling or weighting methods to ensure representation of rural and socioeconomically diverse populations, thereby improving the external validity of the findings. Additionally, health communication campaigns should explicitly test the effectiveness of norm-based and trust-oriented messages in more diverse demographic subgroups to determine the generalizability and efficacy of these strategies across different population segments.

Fourthly, China’s zero-COVID policy and state-controlled media created a unique risk communication environment characterized by institutional trust and social norms, which may limit the generalizability of our findings to other cultural contexts or different types of health risks. However, other collectivist or state-influenced health systems may exhibit similar dynamics. Future comparative studies (e.g., East Asian or Middle Eastern contexts) can explore how institutional trust interacts with social conformity in shaping public health behavior, enhancing the model’s cross-cultural applicability.

Finally, our cross-sectional design captures these relationships at only a single point in time, despite the likely evolution of perceptions and behaviors during a prolonged crisis. Therefore, it may limit our ability to infer causality or capture the temporal dynamics of risk perception and information-seeking during the evolving stages of the COVID-19 pandemic. Future longitudinal studies could better illustrate how these processes unfold over time, particularly as information needs and institutional messages evolve.

## Conclusion

The unprecedented COVID-19 pandemic has presented a formidable global public health challenge, demanding a deeper understanding of public responses and engagement. Drawing on the established RISP (2), this study reveals that while traditional assumptions about the emotional drivers of information-seeking may not hold, social norms and institutional trust play distinct roles in shaping information-seeking behaviors. Our findings demonstrate that uncertainty can enhance perceived control over information seeking, and trust in experts may paradoxically increase risk perception.

While conducted during the COVID-19 pandemic, these insights remain highly relevant for future public health challenges, from emerging infectious diseases to endemic conditions, particularly in contexts where institutional trust and social conformity significantly influence public health behaviors. These findings suggest specific approaches for national health systems and international organizations, such as the WHO. First, leverage social norms through community-based communication strategies rather than relying primarily on emotional appeals. Second, institutional credibility must be maintained through transparent risk communication, which paradoxically may increase risk awareness while maintaining trust. Third, uncertainties should be acknowledged while providing clear information-seeking guidance. These principles are especially relevant for health systems with strong institutional structures, where communication strategies should balance centralized messaging with community-level engagement to maintain public trust during evolving health crises.

## Data Availability

The raw data supporting the conclusions of this article will be made available by the authors, without undue reservation.
